# Research on the influence mechanism and governance mechanism of digital divide for the elderly on wisdom healthcare: The role of artificial intelligence and big data

**DOI:** 10.3389/fpubh.2022.837238

**Published:** 2022-08-17

**Authors:** Jian Zhou, Zeyu Wang, Yang Liu, Jian Yang

**Affiliations:** ^1^Department of Business Management, School of Business, Qingdao University, Qingdao, China; ^2^Department of Business Administration, Edinburgh Business School, Heriot-Watt University, Edinburgh, United Kingdom; ^3^Department of Cultural Industry, Cultural Industry Research Institute, Qilu University of Technology, Jinan, China; ^4^Department of Computer Science and Technology, Ocean University of China, Qingdao, China

**Keywords:** digital divide, older people, wisdom health care, artificial intelligence, big data, governance mechanism

## Abstract

With the rapid development of digital information technology, life has become more convenient for people; however, the digital divide for the elderly was even more serious, so they became a forgotten group in the internet age over time. Residents' demand for healthcare is rising, but the wisdom healthcare service supported by digital information technology is less acceptable to the elderly due to the digital divide. Based on the knowledge gap theory and combining the value perception and satisfaction model, this study explores the influence of the digital divide for the elderly on wisdom healthcare satisfaction and takes the perceived value of wisdom healthcare as a mediator, and artificial intelligence and big data as moderators into the research framework. Based on the data of 1,052 elderly people in China, the results show that the digital divide for the elderly has a negative influence on wisdom healthcare satisfaction and perceived value. Moreover, it is found that wisdom healthcare perception value mediated the relationship between the digital divide for the elderly and the wisdom healthcare satisfaction, which enhances the negative effect of the digital divide for the elderly on wisdom healthcare satisfaction. Furthermore, the moderating effect of artificial intelligence and big data on the relationship between the digital divide for the elderly and the perceived value of wisdom healthcare is opposite to that between the perceived value of wisdom healthcare and wisdom healthcare satisfaction. Therefore, this study has a reference value for the development and optimization of smart medical industry.

## Introduction

Aging population of China is deepening year by year. According to the data of the Seventh National Census, China has gradually entered the moderate aging stage, with about 191 million people aged 65 or above, accounting for 13.5% of the total population. As residents' medical needs are growing and the information age is evolving, a wisdom healthcare service model supported by digital technologies such as internet of things, big data, and cloud computing is emerging at the historic moment (1, 2). The concept of wisdom healthcare comes from intelligent care for aged proposed by the British Life Trust Fund. Specifically, wisdom healthcare refers to be free from time and space limitation of the traditional aged care model, integrating all service participants with the help of modern science and technology, and an organic whole is formed to improve the quality of aged care services, through internet of things platforms such as government, community, and medical institutions. Under the dual background of aging and information society, wisdom healthcare, as a new pension model in the internet age, not only reshaped the aged care model in technology and information but also provided drivers for the value creation and value perception of the pension industry in terms of service (3, 4). However, existing studies have found that neither the content of digital communication nor the design of digital devices have paid attention to the real needs of the elderly; therefore, the elderly have gradually become a forgotten group in the internet age, which inevitably leads to the digital divide for the elderly (5–7). Furthermore, there are serious cognitive deviations and cognitive impairments in the process of receiving wisdom healthcare for the elderly, while the digital divide for the elderly has become an obstacle for them to enjoy wisdom healthcare services (8, 9).

Facing the aging population, the new era characterized by digitalization, networking, and intelligence has provided new technical means for the social communication of the elderly to alleviate the social isolation due to the aging of the elderly (10, 11). However, the shortcomings of digital devices such as technological components, complicated operation interfaces and difficult understanding have further deepened the “digital divide,” resulting in the refusal of the elderly to wisdom healthcare service (12, 13). Therefore, the elderly generally have low participation, recognition, and satisfaction with the wisdom healthcare model, which is not conducive to the further development and optimization of it, as well as to the expansion of academic research in this field (14, 15). With that in mind, in the internet era with the rapid development of digital information, in order to further promote the development and optimization of the wisdom healthcare model and improve the quality of life of the elderly, this study, from the value perspective of wisdom healthcare, discusses the influence of the “digital divide for the elderly” on the value perception and satisfaction of wisdom healthcare, which has crucial theoretical and practical significance.

With the development of information and digital opportunities, digital divide for the elderly, a new social governance issue, has also emerged (16, 17). As for the digital divide, research studies show that it exists not only between countries and regions with different levels of economic development but also between different groups of people in the same region. Different educational backgrounds, occupations, and ages are the main reasons for this phenomenon (18, 19). However, the unsatisfactory physical condition of the elderly, coupled with complex operation and difficult understanding of digital devices, leads to an unfavorable sense of experience for the elderly to use related products and enjoy services, causing instinctive rejection of the internet and digital technology (20). The digital divide for the elderly has greatly affected the value transmission of the resources for them. For example, the “digital access gap” and “digital use gap” directly hinder the acceptance and perception of the value contained in wisdom healthcare service for the elderly, while the “digital knowledge gap” makes the elderly feel more socially isolated and deepens their rejection of wisdom healthcare service (21). The existing research found that the development of digital technologies such as artificial intelligence and big data is beneficial to meet the real needs of the elderly, give reasonable advice, and provide comfortable services. Artificial intelligence and big data have enabled the elderly to “passively” express their real demands, which is conducive to wise decision-making by policymakers of wisdom healthcare and, to a certain extent, can alleviate the negative influence of the digital divide for the elderly on the perception and satisfaction for the value of wisdom healthcare (22–24).

Based on the aforementioned analysis, this study, in the context of rapid development of digital information and the growing demand for the elderly, explores the influence of the digital divide on value perception and satisfaction of wisdom healthcare, as well as the moderating effect of artificial intelligence and big data, according to the knowledge gap theory and the value perception and satisfaction model, and the digital divide, artificial intelligence, big data, perceived value, and satisfaction of wisdom healthcare all contained in the theoretical model. Focusing on the influence mechanism of artificial intelligence on the “digital divide” for the elderly on wisdom healthcare, the research results enrich the related research of the knowledge gap theory, which not only theoretically expands the application scope of the value perception and satisfaction model but also provides a useful reference for the development and optimization of smart medical industry.

The remainder of this article addresses several issues. First, literature review conducted on the digital divide, knowledge gap theory, and the relationship between the “digital divide” for the elderly and the satisfaction of wisdom healthcare is presented, and relevant research hypotheses are proposed. Second, various measures of the study variables and model estimation are introduced. Third, the empirical findings are presented and discussed. Last but not least, the limitations of this study are summarized.

## Theoretical review and hypothesis

### Digital divide and knowledge gap theory

As a social phenomenon caused by the rapid development of digital technology, the digital divide was not originally applied to study the elderly. It is defined as the gap between technology owners and technology users, which means the differences in the accessibility and use of technology due to diverse conditions (25–29). According to the traditional digital divide theory, there are two main reasons for the emergence of it. First, the difference in the material level leads to the digital divide, which is the degree of new technology access caused by economic development, network facility construction, and network rules. This is also known as the access gap of the digital divide due to unequal regulations (25, 30, 31). Second, the differences in the application level also give rise to the digital divide, which is the differences in the ways and degrees of using new technologies due to the level of literacy, digital skills, and habits of different groups, also known as the use gap of the digital divide (32, 33). In addition to the access gap and use gap, researchers of the knowledge gap theory also found that there were significant differences in the acquisition and use of internet knowledge among different groups during internet access, which are caused by differences in economic status and resource endowments among different groups, also known as the knowledge gap of the digital divide (34–36).

The research found that in the era of digitalization and aging, the use of digital technology by the elderly lags far behind that of the young due to the subjective distrust and information rejection of the elderly, as well as the objective physical function and social neglect (37, 38). According to the WeChat usage report released by Tencent Research Institute, the average length of time that older people spend on WeChat per day, the number of functions they master, and the number of friends they have on WeChat are significantly lower than those of young people (39). Especially due to the information spillover effect brought by the rapid development of digital technology, the digital divide between the elderly and the young tends to further expand, which is caused by the differences of the social and economic status and cognitive processing ability of the elderly themselves (40, 41), that is, the social and economic status of the elderly and the use of digital technology online are linked. In addition, with the decline of physiological functions of the elderly, their cognitive ability and reprocessing ability of digital information will further widen the gap with the young (42, 43). Therefore, it is necessary to reasonably analyze the causes of the digital divide for the elderly, as well as its influence mechanism and governance mechanism.

### Digital divide of the elderly and the satisfaction of wisdom healthcare

Wisdom healthcare is a new modern service model for the aged, which is characterized by intelligence, individuality, diversification, and information interconnection. It came into being with the development of digital technologies such as artificial intelligence, big data, and internet of things in the digital information era (44, 45). Wisdom healthcare not only subverts the traditional aged care model from the technical aspect but also provides new development requirements for the value creation and value perception of the aged care industry from the service aspect (46). The popularization and application of digital technology make the life of the elderly more intelligent, convenient, and happy (47). However, the digital divide for the elderly caused by the strong rejection of network information and the unskilled use of digital technology makes the wisdom healthcare become unacceptable by the elderly and even produces completely negative experience.

The influence of the digital divide for the elderly on the satisfaction of wisdom healthcare is mainly reflected in the following points: First, in terms of the digital access gap, as teenagers have become the main users of digital information technology, the design and communication contents of major media mainly focus on the preferences of teenagers, while the needs of the elderly are neglected; therefore, information technology to adapt to the aging is relatively insufficient (48). The complicated rules and procedures of using internet and digital technology lead to the instinctive rejection of digital information by the elderly, and their disconnection with digital information technology makes it difficult for the elderly to integrate into the wisdom healthcare mode, resulting in lower satisfaction with wisdom healthcare. Second, in terms of digital usage gap, the decline of cognitive and processing ability, memory, and other physical functions makes the elderly unable to skillfully use digital information devices. It is challenging for the elderly to use smart devices with small buttons and complicated operation procedures; therefore, they have negative emotions about the wisdom healthcare model, which reduce the satisfaction of it. Finally, in terms of the digital knowledge gap, people of higher social status can acquire knowledge and information through information technologies such as the internet more quickly and efficiently than people of lower social status (49), while the social and economic status of the elderly may decline after retirement, and they will inevitably be at a disadvantage in acquisition of knowledge (50). Furthermore, some scholars believed that the greatest benefits brought by knowledge will be attributed to those with higher social and economic status (51); therefore, it is difficult for the elderly of lower socioeconomic status to get a positive feedback from the use of knowledge. As a result, the wisdom healthcare model relying on the internet and big data is significantly challenged for the elderly with digital knowledge gap, which directly leads to the lower satisfaction of wisdom healthcare. Based on this, the following hypotheses are proposed in this article:

H1: The digital divide has a negative influence on the satisfaction of wisdom healthcare.
H1a: The digital access gap has a negative influence on the satisfaction of wisdom healthcare.
H1b: The digital usage gap has a negative influence on the satisfaction of wisdom healthcare.
H1c: The digital knowledge gap has a negative influence on the satisfaction of wisdom healthcare.

### The mediating role of wisdom healthcare value perception

Customer value proposition and value perception will have a significant impact on their consumption behavior (52, 53). When customers feel the unique value of products or services, they will have the psychological state of “customer loyalty,” while customer preference leads to consumers' lasting desire and behavior of purchasing (54–56). The aged care service provided by wisdom healthcare is a typical consumer service product, so the perceived value of the elderly is one of the key factors to decide whether choose this service. According to the existing research on the value perception of wisdom healthcare, value perception of the elderly is mainly divided into five categories, namely, functional value, cognitive value, social value, emotional value, and conditional value.

In terms of the functional value perception of wisdom healthcare, as the result of the digital divide and the use gap in particular, the elderly are unable to skillfully use smart devices for wisdom healthcare services and have poor experience in it, so they cannot realize the functional value of wisdom healthcare. As a result, they cannot perceive the unique value of service products, causing a lower satisfaction level of wisdom healthcare (20, 21). In terms of cognitive value perception of wisdom healthcare, the content of mainstream media and the design of devices seldom take into account the real needs of the elderly. As a group that has been “forgotten” by the internet, the elderly cannot be as “handy” as the young in acquiring internet information or using digital devices, and there are serious digital access gaps, digital use gaps, and digital knowledge gaps (7, 42); therefore, it is difficult for the elderly to feel the spiritual and humanistic care, which is closely related to the cognitive value in value perception. Consequently, with the decrease in cognitive value perception, the satisfaction of wisdom healthcare is also lower. In terms of social value perception of wisdom healthcare, as the existence of the digital divide for the elderly, they seldom perceive the group norms and social identity while enjoying the services provided by wisdom healthcare; thus, without a sustained desire to purchase, the satisfaction of wisdom healthcare declines (57). In terms of emotional value perception of wisdom healthcare, the digital divide for the elderly leads to communication obstacles within the elderly group and between the elderly and the young, and the deterioration of interpersonal relationships, resulting in much less willingness to communicate with others and less emotional value perception of wisdom healthcare services, which further reduce the satisfaction of wisdom healthcare. In terms of conditional value perception of wisdom healthcare, due to the digital divide, the elderly cannot obtain conditional preference when enjoying the services provided by wisdom healthcare, so they cannot perceive the conditional value contained in wisdom healthcare services through unique conditional preference, which further reduces the satisfaction of wisdom healthcare (58). Based on this, the following hypotheses are proposed:

H2: Perceived value of wisdom healthcare mediates the relationship between the digital divide and the satisfaction of wisdom healthcare.
H2a: Perceived value of wisdom healthcare mediates the relationship between the digital access gap and the satisfaction of wisdom healthcare.
H2b: Perceived value of wisdom healthcare mediates the relationship between the digital use gap and the satisfaction of wisdom healthcare.
H2c: Perceived value of wisdom healthcare mediates the relationship between the digital knowledge gap and the satisfaction of wisdom healthcare.

### Governance mechanism of artificial intelligence and big data

Artificial intelligence and big data are gradually applied to medical services with better extensibility, faster processing capacity, and higher reliability (59–61). The application of artificial intelligence and big data in wisdom healthcare services mainly involves three levels: the molecular level, mainly used for the research of drugs and healthcare products, as well as research, development, and production of related drugs according to the physical conditions of the elderly; the clinical level, which improves the accuracy of clinical data assessment and accurately analyzes the physical function of the elderly to take reasonable treatment; and the social level, mainly used for informatics research to prevent the emergence of large-scale epidemics by tracking and analyzing big data (62).

It is found that the application of artificial intelligence and big data contributes to the development of healthcare services for the elderly and to the prevention, diagnosis, treatment, and management decision of future public health events (63). The complexity of big data analysis lies in the integration of all kinds of information and the transformation of large amounts of data into operational knowledge of precise medicine for decision makers. Moreover, artificial intelligence is faster and more accurate than medical staff and has performed well-in clinical treatment and rehabilitation. Artificial intelligence and big data provide a lot of convenience for medical services, especially for the elderly, that is, whether in the stage of prevention, treatment, or rehabilitation, products and services provided by wisdom healthcare are more efficient, diversified, and easily accepted by the elderly. Undoubtedly, with artificial intelligence and big data, on the one hand, more informed decisions for wisdom healthcare can be made by policymakers according to the unique situation of the elderly so as to improve the management efficiency of the government; on the other hand, more accurate information about the needs of the elderly should be obtained to provide more targeted services by wisdom healthcare, thus improving the possibility for the elderly to perceive the value of wisdom healthcare and weakening the negative effect of the digital divide for the elderly. Meanwhile, the application of artificial intelligence and big data has increased the society's understanding of the elderly. As for high-quality services and cultural and entertainment products for the elderly, the services provided by wisdom healthcare are more targeted and more easily accepted by the elderly population, thus improving the satisfaction level of it. Therefore, the application of artificial intelligence and big data has enhanced the positive impact of the perceived value of wisdom healthcare on the satisfaction of wisdom healthcare. Based on this, the following hypotheses are proposed:

H3: Artificial intelligence and big data negatively moderate the relationship between digital divide and perceived value of wisdom healthcare, that is, the negative relationship is stronger when artificial intelligence and big data are at a low level.
H4: Artificial intelligence and big data positively moderate the relationship between perceived value of wisdom healthcare and satisfaction of wisdom healthcare, that is, the positive relationship is stronger when artificial intelligence and big data are at a high level.

**Figure 1:**
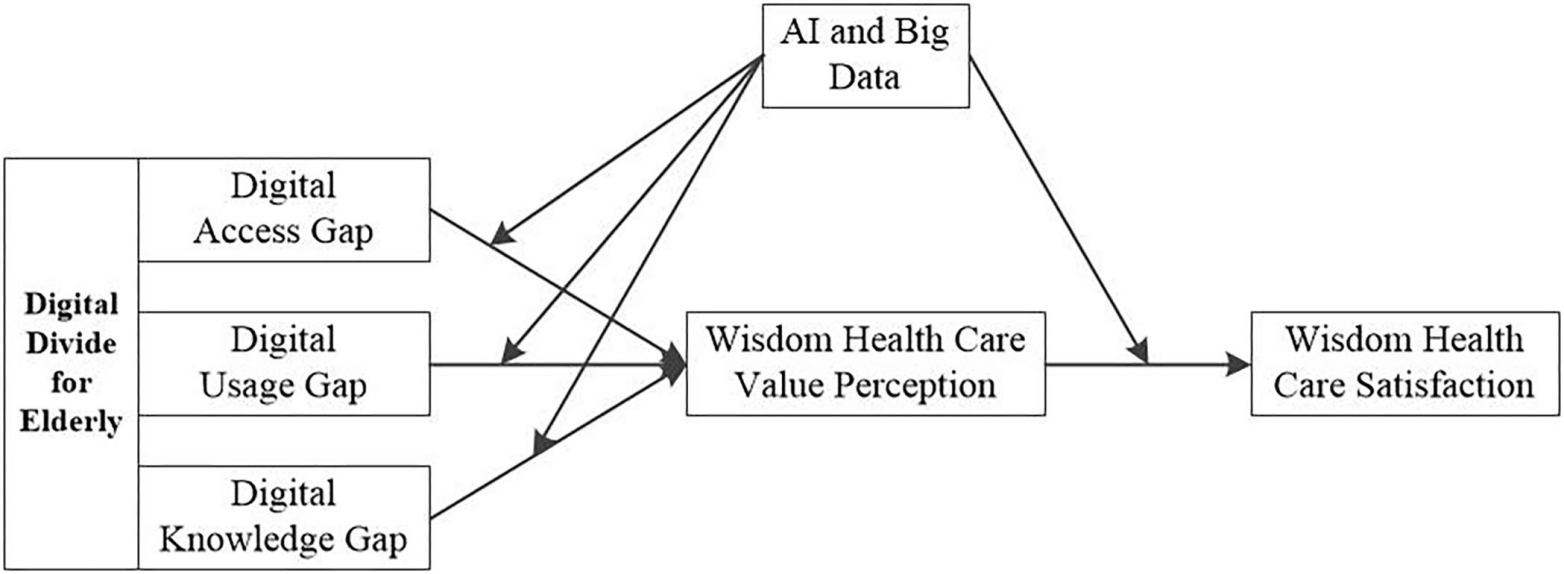
Research model.

The conceptual framework for this study is shown in Figure 1.

## Materials and methods

### Sample and procedures

As the research content of this study mainly involves the “digital divide” and “wisdom healthcare” -related topics of the elderly, the research object is the elderly group, and those who have enjoyed or experienced the related wisdom healthcare services were selected in the specific investigation process. In the process of data collection, it is found that the elderly generally have poor comprehension ability, slow self-reading speed, and poor eyesight, so the traditional questionnaire distribution is not applicable. Therefore, the research group adopted the interview questionnaire to collect data and went deep into the typical areas where the elderly gather, such as smart healthcare communities, smart healthcare industrial parks, and smart healthcare towns. These areas characterized by a large number of smart healthcare devices, such as smart beds, smart toilets, wearable intelligent monitoring devices, and medical auxiliary devices. At the same time, in order to avoid common methodological biases and other problems, data collection was conducted twice in July 2021 and September 2021. In addition, in the process of data collection, the staff of neighborhood committees were invited to participate, which, to a large extent, eliminated the concerns of the elderly to participate in this survey. In this study, about 1,200 questionnaires were sent out for the elderly in these communities. After careful screening and identification, a total of 1,052 valid questionnaires were collected, with an effective recovery rate of 87.67%.

### Variable measurement

For the digital access gap, the virtual variable is online is taken as the measurement index (online = 1, no online = 0), referring to the research results of Hao et al. (64). For the digital use gap, referring to the research results of Zeng et al. (65), it is measured from two dimensions with a scale of six items, that is, the time and purpose of the elderly to use the internet, which mainly include the average daily online time of the elderly and whether they can obtain the knowledge related to life, consumption, and healthcare on the internet. For the digital knowledge gap, it is measured by a scale of five items, referring to the research of Scheerder et al. (66). Since this study focuses on the influence of the digital divide on wisdom healthcare, the digital knowledge gap mainly measures the knowledge of the elderly about the wisdom healthcare, mainly including the concept, function, type, and understanding.

As for artificial intelligence and big data, referring to the research results of Yao et al. (67), there are typical questions such as “What do you think whether artificial intelligence and big data can help ease the digital divide and understand the wisdom healthcare?”.

Referring to the research results of Flanagin et al. (68), the perceived value of wisdom healthcare is measured with a scale of five items, which mainly include the functional value, the cognitive value, the social value, the emotional value, and the conditional value.

Referring to the research results of Hightower et al. (69) and Mattila et al. (70), the satisfaction of wisdom healthcare is also measured with a scale of five items, mainly including happiness of the elderly when receiving services, their willingness to continue receiving services, their praise and gratitude to wisdom healthcare institutions, and the direct satisfaction evaluation of wisdom healthcare services.

With reference to previous research results, the individual characteristics of the elderly receiving services are selected as control variables, mainly including gender, age, education level, and income level.

### Common method bias

Aiming at the common methodological bias in the process of data collection, the research team of this study divided the questionnaire into two parts according to the research questions. Furthermore, data collection was carried out in July 2021 and September 2021, respectively, so as to reduce such problems by time interval. Because of the memory gap between the elderly and the young, the time interval of 2 months is considered appropriate. At the same time, in order to further verify the quality of the questionnaire, this study uses the Harman single-factor test to evaluate the common methodological bias of the main variables involved and to make principal component analysis of the research items involved in the previous theoretical model of this study without rotation. The results show that the first factor without rotation can explain 29.66% variance, while the cumulative variance can explain 65.59% variance; thus, the variance explained by the first factor is less than half of the total explained variance. In addition, we use the marker variable method to test the CMV and choose pro-social motivation as a marker variable, which contains four items. It is found that the chi-square value does not change significantly after the marker variable is added, so it can be considered that there is no serious common methodological bias in this study.

## Results

### Confirmatory factor analysis

In this study, SPSS22.0 and Mplus7.0 software packages were used for confirmatory factor analysis, as shown in Table 1. The six-factor model has the best fitting degree with the data (χ2 (253)/DF = 1.57, RMSEA = 0.06, CFI = 0.95, TLI = 0.94, SRMR = 0.04). Furthermore, through comparative analysis, it is found that the model constructed in this study is superior to other alternative models, which shows that the six-factor model meets the standards of model construction and analysis (71).

**Table 1:** Results of confirmatory factor analysis.

	**χ2**	**Df**	**χ2 /Df**	**RMSEA**	**CFI**	**TLI**	**SRMR**
Six-factor model	396.73	253	1.57	0.06	0.95	0.94	0.04
Four factors model^a^	653.76	228	2.87	0.08	0.89	0.90	0.05
Three factors model^b^	1053.76	227	4.64	0.08	0.80	0.78	0.06
Two factor model^c^	1172.91	229	5.12	0.09	0.77	0.78	0.07
Single factor model^d^	1272.91	230	5.53	0.09	0.66	0.74	0.09

n = 1,052.

^a^Combine the digital divide into a potential factor.

^b^Combine the digital divide and artificial intelligence as a potential factor.

^c^Combine the digital divide, artificial intelligence and wisdom health value perception into a potential factor.

^d^Attributing all items to the same potential factor.

### Descriptive statistical analysis

The mean, standard deviation, and correlation coefficient of the main variables in this study are shown in Table 2. The data show that the digital access gap (*r* = −0.58, *p* < 0.01), digital use gap (*r* = −0.48, *p* < 0.01) and digital knowledge gap (*r* = −0.63, *p* < 0.01), digital use gap (*r* = −0.54, *p* < 0.01), and digital knowledge gap (*r* = −0.57, *p* < 0.01) are also negatively correlated with the perceived value of wisdom healthcare, and there is a negative correlation between the perceived value of wisdom healthcare and the satisfaction of wisdom healthcare (*r* = 0.62, *p* < 0.01).

**Table 2:** Means, standard deviations, and correlations.

**Variable**	**1**	**2**	**3**	**4**	**5**	**6**	**7**	**8**	**9**	**10**
1. Gender	1									
2. Age	−0.11**	1								
3. Education	−0.08*	0.32**	1							
4. Income level	−0.07	0.37**	0.01	1						
5. Digital access gap	0.03	0.07	0.18**	−0.21**	1					
6. Digital usage gap	0.10*	−0.11*	0.06	−0.02	0.64**	1				
7. Digital knowledge gap	0.04	0.04	0.23**	−0.29**	0.61**	0.50**	1			
8. AI and Big Data	0.07	−0.10	0.11*	0.14*	−0.47**	−0.60**	−0.64**	1		
9. Wisdom health care perception value	0.06	−0.04	0.12*	0.06	−0.49**	−0.54**	−0.57**	0.59**	1	
10. Wisdom health care satisfaction	0.05	0.05	0.26**	−0.22*	−0.58**	−0.48**	−0.63**	0.63**	0.62**	1
Cronbach's α	N/A	N/A	N/A	N/A	0.74	0.83	0.83	0.94	0.78	0.85
Mean	1.63	2.69	2.77	3.63	4.24	4.26	4.17	4.95	5.37	5.53
Standard deviation (SD)	0.48	1.80	2.09	2.66	1.33	1.35	2.01	1.02	1.19	1.24

n = 1,052, *Significant at the p < 0.05 (**p < 0.01) level. N/A indicates not suitable for analysis.

### Hypothesis testing

The data of the main effect hypothesis H1 and its sub-hypotheses are tested using the hierarchical regression. Among them, M4, as the benchmark model, represents the influence of gender, age, educational background, and income level of the elderly on the satisfaction of wisdom healthcare. M5 shows that based on the benchmark model, digital access channel, digital use channel, and digital knowledge channel are added into the model by regression. The results show that three independent variables can significantly affect the satisfaction of wisdom healthcare (M5: β1 = −0.51, β2 = −0.38, β3 = −0.40, *p* < 0.01). Therefore, the three main effect hypotheses proposed in this article are all supported.

On the basis of principal effect analysis, the mediation effect hypothesis H2 and its sub-hypotheses proposed in this article are analyzed in three steps. The first step refers to M2 in Table 3, indicating that digital access gap, digital use gap, and digital knowledge gap can significantly affect the perceived value of wisdom healthcare (M2: β1 = −0.54, β2 = −0.49, β3 = −0.45, *p* < 0.01). The second step refers to M6 in Table 3, indicating that the perceived value of wisdom healthcare can significantly affect satisfaction of wisdom healthcare (M6: β = 0.37, *P* < 0.01). The third step refers to M7 in Table 3, which shows that after digital access gap, digital use gap, digital knowledge gap, and wisdom healthcare perception value are simultaneously included in the regression model; the influence of the three independent variables on the satisfaction of wisdom healthcare is significantly reduced ((M7: β1 = −0.27, β2 = −0.31, β3 = −0.19, *P* < 0.01), while the relationship between the perceived value of wisdom healthcare and satisfaction of wisdom healthcare is still significant (M7: β = 0.32, *P* < 0.01), which shows that the hypothesis of the mediation effect was supported.

**Table 3:** Hierarchical regression results.

**Explanatory variable**	**Wisdom health care perception value**			**Wisdom health care satisfaction**				
	**M1**	**M2**	**M3**	**M4**	**M5**	**M6**	**M7**	**M8**
Gender	0.02	0.13	−0.02	0.05	0.04	0.01	0.03	0.03
Age	−0.08	−0.04	0.00	−0.05	−0.01	0.03	0.02	0.02
Education	0.26**	0.14**	0.20**	0.28**	0.17**	0.23**	0.07**	0.07*
Income level	−0.33**	−0.96**	−0.29**	−0.24**	−0.11**	−0.20	0.03	0.04
Digital access gap		−0.54**	−0.50**		−0.51**		−0.27**	−0.29**
Digital usage gap		−0.49**	−0.44**		−0.38**		−0.31**	−0.30**
Digital knowledge gap		−0.45**	−0.41**		−0.40**		−0.19**	−0.22**
Wisdom health care perception value						0.37**	0.32**	0.30**
AI and big data			0.16*					0.19*
Interactive 1			−0.35**					
Interactive 2			−0.39**					
Interactive 3			−0.51**					
Interactive 4								0.22**
*R*2	0.15	0.41	0.37	0.12	0.37	0.31	0.59	0.70
Δ*R*2	0.15	0.26	0.22	0.12	0.25	0.19	0.47	0.58
*F*	24.74	267.75**	69.25**	20.05	68.84**	53.61**	254.18**	220.56**
Δ*F*	24.74	118.56**	211.36**	20.05	232.03**	165.16**	946.03**	614.09**

n = 1,052, **p < 0.01, *p < 0.05. Interactive 1 = digital access gap × AI and big data, Interactive 2 = digital usage gap × AI and big data, Interactive 3 = digital knowledge gap × AI and big data, Interactive 4 = wisdom health care value perception × AI and big data.

In total, two steps are used to analyze the moderating effect hypotheses H3 and H4 proposed in this article. The first step refers to M3 in Table 3, indicating the moderating effect of artificial intelligence and big data on the relationship between three independent variables and perceived value of wisdom healthcare (M3: β1 = −0.35, β2 = −0.39, β3 = −0.51, *P* < 0.01). The second step refers to M8 in Table 3, indicating the moderating effect of artificial intelligence and big data on perceived value of wisdom healthcare and satisfaction of wisdom healthcare (M8: β = 0.22, *P* < 0.01), which shows that the moderating effects are supported.

## Conclusion and implications

### Conclusion

Based on the knowledge gap theory, this study explores the relationship among the digital divide, perceived value of wisdom healthcare, artificial intelligence, big data, and satisfaction of wisdom healthcare through the empirical analysis of 1,052 valid questionnaire data. The results are as follows: First, the digital divide has a negative effect on satisfaction of wisdom healthcare. In terms of the digital access gap, the elderly are not the target audience of digital information technology, leading to disconnection between the elderly and the wisdom healthcare. In terms of the digital access gap, the elderly are unable to skillfully operate the relevant digital devices due to their physical functions, resulting in negative emotions. In terms of the digital knowledge gap, as the knowledge acquisition ability of the elderly declines, it is difficult to obtain the positive feedback of use, thus reducing their satisfaction of the wisdom healthcare. Second, the perceived value of wisdom healthcare plays a mediated role between the digital divide and satisfaction of wisdom healthcare. Due to the digital access gap, usage gap, and knowledge gap, the elderly cannot perceive the cognition and experience brought by wisdom healthcare, which reduces the perceived value of wisdom healthcare and thus affect their satisfaction of wisdom healthcare. Third, artificial intelligence and big data negatively moderate the relationship between the digital divide for the elderly and the perceived value of wisdom healthcare, while they positively moderate the relationship between the perceived value of wisdom healthcare and satisfaction of wisdom healthcare. The application of artificial intelligence and big data will provide accurate medical care and convenient and efficient services for the elderly so as to improve their perception of wisdom healthcare and weaken the negative effects of the digital divide for the elderly. Meanwhile, artificial intelligence and big data will increase the society's understanding of the elderly, provide targeted services, quantify the perceived value of wisdom healthcare, and improve the satisfaction level of wisdom healthcare.

### Theoretical implications

This study enriches the research on the digital divide of the elderly and wisdom healthcare satisfaction and has certain theoretical implications in this field, which are embodied in the following three aspects:

First, existing research has paid little attention to the digital divide and wisdom healthcare. Existing literature has made a preliminary discussion on the digital divide of the elderly from some fields and perspectives, such as enhancing their own health management, promoting the formation of positive mentality, and making it easier to obtain health information. However, few studies have systematically studied the digital divide from the perspective of the influencing mechanism of wisdom healthcare, and this study has filled the gap in this field.

Second, by excavating the inherent law of the influence of the digital divide on wisdom healthcare of the elderly, this study can not only examine the obstacles of wisdom healthcare from the theoretical point of view under the background of aging population but also seek effective measures to cross the digital divide, find ways to improve wisdom healthcare, creatively combine the digital divide of the elderly with the field of wisdom healthcare, and enrich the theoretical research results in related fields.

Third, this study provides a new research perspective for the new problems in the process of accelerating aging in China and analyzes the influence mechanism of “digital divide” on the wisdom and health of the elderly in the internet and information age. The research conclusion will enrich the policy design of the elderly group, which is the new development of aging economics.

### Practical implications

The results of this study have practical significance for facing up to the “digital divide” for the elderly and improving the satisfaction of wisdom healthcare, which are embodied in the following three aspects:

First, through a questionnaire survey of the elderly, the inherent law and clear influencing mechanism from the digital divide to the satisfaction degree of wisdom healthcare are found so as to explore obstacles of wisdom healthcare and effective intervention schemes to seek effective measures to bridge the “digital divide” under the background of population aging. Second, this study explores the effective ways to improve the quality of wisdom healthcare for the elderly and their happiness and discusses ways to improve the quality of wisdom healthcare for the elderly. Furthermore, there are deep understandings of this issue in the distinctions, the development ways, and the influencing mechanisms of intervention factors and effects. Third, research on the influence mechanism of artificial intelligence and big data on the digital divide and wisdom healthcare is beneficial for the government to formulate relevant policies and cooperate with all sectors of society to increase exchanges in infrastructure investment to implement relevant policies.

### Limitations and future directions

However, there are some limitations to this study, which can be used for reference in future research. First of all, as scholars pay little attention to this field, there are limited results that can be directly used for reference in combination with national conditions; therefore, the subsequent research can consider the theoretical constructive design driven by the aging of national conditions and explore more crucial issues from the perspective of satisfaction of wisdom healthcare. Furthermore, although some measures have been taken to improve the representativeness and accuracy of the data source, the overall data are still sectional data with certain limitations. Hence, in subsequent studies, randomized controlled trials should be introduced to strengthen the external validity and universality of the conclusion. Finally, the satisfaction of the elderly with digital divide on wisdom healthcare needs to be further studied from multiple dimensions, that is, future research should further analyze the phenomenon of “digital divide” and its differences among the related and different groups of the elderly, the influencing factors of the digital divide, and its correlation and influencing mechanism on the smart devices related to wisdom healthcare.

## Data availability statement

The raw data supporting the conclusions of this article will be made available by the authors, without undue reservation.

## Ethics statement

Ethical review and approval was not required for the study on human participants in accordance with the local legislation and institutional requirements. Written informed consent for participation was not required for this study in accordance with the national legislation and the institutional requirements.

## Author contributions

Conceptualization and validation: JZ and YL. Data curation, methodology, software, and supervision: JY. Formal analysis: JZ and JY. Funding acquisition, investigation, project administration, resources, visualization, writing—original draft, and writing—review and editing: JZ and ZW. All authors have read and agreed to the published version of the manuscript.

## Funding

This research was partly funded by the Shandong Province Social Science Planning Research Project (21CSHJ03).

## Conflict of interest

The authors declare that the research was conducted in the absence of any commercial or financial relationships that could be construed as a potential conflict of interest.

## Publisher's note

All claims expressed in this article are solely those of the authors and do not necessarily represent those of their affiliated organizations, or those of the publisher, the editors and the reviewers. Any product that may be evaluated in this article, or claim that may be made by its manufacturer, is not guaranteed or endorsed by the publisher.
